# Lipid nanoparticle-formulated DNA acts as a potent immune modulator for cancer immunotherapy through interferon signaling pathways

**DOI:** 10.7150/thno.121364

**Published:** 2026-01-01

**Authors:** Chen-Yi Chiang, Ming-Shu Hsieh, Mei-Yu Chen, Yu-Wen Tsai, Chang-Ling Lin, Chia-Wei Hsu, Guann-Yi Yu, Ming-Hsi Huang, Shih-Jen Liu, Hsin-Wei Chen

**Affiliations:** 1 National Institute of Infectious Diseases and Vaccinology, National Health Research Institutes, Miaoli, Taiwan.; 2 Graduate Institute of Biomedical Sciences, China Medical University, Taichung, Taiwan.; 3 Graduate Institute of Medicine, Kaohsiung Medical University, Kaohsiung, Taiwan.

**Keywords:** immunotherapy, inflammatory responses, lipid nanoparticles, plasmid DNA, tumor microenvironments

## Abstract

**Rationale:** Plasmid DNA (pDNA) delivered by lipid nanoparticles (LNPs) represents a promising strategy for cancer immunotherapy, offering both stability of nucleic acids and efficient intracellular delivery. This study aimed to evaluate the stability and immunotherapeutic potential of LNP/pDNA formulations and to define the mechanisms underlying their antitumor activity.

**Methods:** LNP/pDNA complexes were prepared by a microfluidic mixer system. Encapsulation efficiency, particle size, and transfection capacity were determined at different time points following formulation to assess physicochemical stability. *In vivo* antitumor efficacy was evaluated using intratumoral and intramuscular administration in murine tumor models. Mechanistic studies included cytokine profiling, transcriptomic analysis of tumors, and immune cell depletion experiments. Mouse models deficient in TLR9 and interferon signaling pathways were employed to dissect signaling pathway contributions.

**Results:** LNP/pDNA formulations retained encapsulation efficiency and size uniformity after prolonged storage and maintained effective gene delivery. Both intratumoral and intramuscular administration suppressed tumor growth, with local delivery showing superior efficacy. LNP/pDNA activated cytosolic DNA-sensing pathways and induced robust proinflammatory cytokine production. Transcriptomic analysis revealed strong type I and II interferon responses and upregulation of immune effector pathways. Depletion studies confirmed that antitumor effects were dependent on CD8⁺ T cells and NK cells but independent of neutrophils and monocytes. Notably, therapeutic efficacy was preserved in TLR9-deficient mice but lost in mice lacking both type I and II interferon signaling.

**Conclusions:** LNP/pDNA induces potent antitumor immunity through activation of IFN-dependent, TLR9-independent pathways, engaging both innate and adaptive immune responses. These findings support LNP/pDNA as a stable, effective platform for cancer immunotherapy.

## Introduction

Immunotherapy harnesses the host immune system by modulating its activity to eliminate tumor cells, transforming the landscape of cancer treatment 1-4. There is growing interest in nucleic acid-based immunotherapies, including antisense oligonucleotides, small interfering RNAs, short hairpin RNAs, messenger RNAs (mRNAs), and plasmid DNAs (pDNAs). Nucleic acid-based strategies are being applied not only in cancer immunotherapy but also in gene therapy, vaccine development, and regenerative medicine 5-9. These nucleic acid modalities hold broad therapeutic promise due to their favorable efficacy, low toxicity, and cost-efficient production. However, their clinical translation has been hampered by challenges in stability, delivery efficiency, and off-target immunogenicity.

Lipid nanoparticles (LNPs) have emerged as a clinically validated platform for nucleic acid delivery, most notably demonstrated by the success of LNP-based mRNA vaccines against SARS-CoV-2 10-14. LNPs are typically composed of four lipid components. These include ionizable lipids, phospholipids, cholesterol, and PEGylated lipids (PEG-lipids). Several studies have shown that LNP-encapsulated nucleic acids are efficiently delivered into cells and are safe for use *in vitro* and *in vivo* 6, 15-18. Notably, ionizable lipids used in LNPs have been found to possess inherent immunostimulatory properties 19, and LNPs themselves can exhibit adjuvant activity 20-22. Recent studies have systematically screened structurally diverse ionizable lipid libraries and demonstrated that rationally designed ionizable lipid-based LNPs can serve as effective adjuvants to enhance the immunogenicity of subunit vaccines 23. Similarly, formulations using varying ratios of ionizable lipids were shown to exhibit distinct adjuvant activities 24. Collectively, these findings support that LNPs act not only as delivery platforms but also as adjuvant platforms with intrinsic immune-activating potential.

While mRNA-based LNP formulations are immunogenic and effective, they are inherently unstable and typically require ultra-cold storage, limiting their global scalability and accessibility 16, 25. In contrast, plasmid DNA offers advantages such as greater chemical stability, cost-effective large-scale production, and long-term storage at standard refrigeration temperatures.

Intratumoral delivery of immune-modulating agents—including nucleic acids, cytokines, antibodies, dendritic cells, and oncolytic viruses—is under active investigation in both preclinical and clinical settings 26-29. Localized delivery not only enhances antitumor immune responses at the tumor site but also reduces systemic toxicity. Recently, non-viral platforms for nucleic acid delivery have emerged as powerful tools for inducing antitumor immunity and tumor regression 30-33. LNP-based delivery systems have become a leading non-viral approach for nucleic acid delivery.

In this study, we investigate the physicochemical stability, transfection efficiency, and immunotherapeutic potential of LNP/pDNA formulations, including non-coding pDNA formulations lacking immunostimulatory transgenes. We demonstrate that LNP/pDNA retains functional activity after long-term storage and induces robust antitumor effects in murine tumor models. Mechanistic studies reveal that LNP/pDNA activates cytosolic DNA-sensing pathways and promotes the infiltration and activation of cytotoxic immune cells. Importantly, we show that antitumor effects are mediated by both innate and adaptive immune mechanisms and occur independently of Toll-like receptor 9 (TLR9) signaling. Collectively, our findings establish LNP/pDNA as a stable, versatile, and effective platform for cancer immunotherapy and highlight its potential as an alternative to mRNA-based therapeutics.

## Methods

### Plasmid construction

The CBGr99 gene was subcloned into the clinically utilized pVAX1 vector (Thermo Fisher Scientific) with a Kozak sequence added at the 5' end, as previously described 18. The resulting plasmid, pVAX1-CBGr99 (pCBGr99), encodes luciferase and was used as a reporter to evaluate the transfection efficiency and gene expression of LNP/pDNA formulations. Plasmids encoding the costimulatory molecules OX40L and 4-1BBL were generated by cloning their respective sequences into the NheI and XhoI sites of the pVAX1 vector, yielding pOX40L and p4-1BBL. All plasmids were amplified in *Escherichia coli* DH5α cells (ECOS101, Yeastern Biotech, Taipei, Taiwan), followed by purification using an endotoxin-free Qiagen column system (Qiagen, Hilden, Germany).

### LNP/pDNA preparation

Cholesterol was obtained from Sigma-Aldrich (St. Louis). SM-102 (1-octylnonyl ester in chloroform) and 1,2-dimyristoyl-rac-glycero-3-methoxypolyethylene glycol-2000 (DMG-PEG) were obtained from Cayman Chemical Company (Ann Arbor). 1,2-Distearoyl-sn-glycero-3-phosphocholine (DSPC) was purchased from Avanti Polar Lipids (Birmingham). Lipids (SM-102:DSPC:cholesterol:DMG-PEG) were mixed in ethanol at a molar ratio of 50:10:38.5:1.5. Plasmid DNA was dissolved in 25 mM sodium acetate buffer (pH 5.0; EMD Millipore, Burlington). The lipid and DNA solutions were 1:3 (v/v) using a microfluidic mixer system (Precision Nanosystems, Vancouver) to form LNP/pDNA complexes. The resulting formulations were diluted 40-fold in 20 mM Tris buffer (pH 7.2), concentrated using Amicon ultracentrifugal filters (EMD Millipore), and passed through a 0.45-µm filter prior to administration. Final LNP/pDNA preparations were characterized for encapsulation efficiency, particle size distribution, polydispersity index, and transgene expression.

### LNP/pDNA characterization

Particle size and polydispersity index (PDI) were measured by dynamic light scattering (DLS) using a Malvern instrument. Results were reported as z-average particle size (diameter, nm). Encapsulation efficiency (EE%) of DNA in LNPs was calculated using the formula: EE% = [(D₀ - D₁) / D₀] × 100, where D₀ is the total DNA amount before LNP lysis and D₁ is the free (unencapsulated) DNA remaining in solution after lysis.

### Mice

C57BL/6 mice were obtained from the National Laboratory Animal Breeding and Research Center (Taipei, Taiwan). Interferon-α/β receptor-knockout (AB6), interferon-γ receptor-knockout (GB6), combined interferon-α/β and -γ receptor-knockout (AGB6), and TLR9-knockout mice were bred at the Laboratory Animal Center of the National Health Research Institutes (NHRI). All knockout strains shared an identical congenic background with C57BL/6 mice. Mice aged 6 to 10 weeks were used, with 4-8 mice per group. All animals were housed at the NHRI Laboratory Animal Center. All animal procedures were approved by and conducted in accordance with the guidelines of the Institutional Animal Care and Use Committee (IACUC) of NHRI (NHRI-IACUC-108157 and NHRI-IACUC-110125).

### Tumor model

B16F10 melanoma cells (Bioresource Collection and Research Center, Taiwan; BCRC-60031) were cultured in DMEM supplemented with 5% (v/v) heat-inactivated fetal bovine serum (GIBCO), 2 mM L-glutamine (GIBCO), 1 mM sodium pyruvate (GIBCO), and 50 units mL-1 penicillin-streptomycin (GIBCO) at 37 °C under 5% CO₂. Cells were harvested and washed with phosphate-buffered saline (PBS). Anesthetized mice were subcutaneously inoculated in the left flank with 1 × 10⁵ B16F10 cells suspended in 0.1 mL serum-free DMEM. On days 7, 9, and 11, anesthetized mice were administered 50 μL of solutions containing either 1, 10, 100, or 1000 fmol pDNA (~0.002, 0.02, 0.2, or 2 μg pDNA, respectively), formulated as LNP/pNC, LNP/pOX40L, LNP/p4-1BBL, pDNA alone, or empty LNP. For certain experiments, mice were intraperitoneally administered 250 μg per mouse of purified antibodies, including anti-CD8 (Ultra-LEAF™, 53-6.7, BioLegend), anti-NK1.1 (Ultra-LEAF™, PK136, BioLegend), and anti-Ly6G (Ultra-LEAF™, 1A8, BioLegend), to deplete CD8⁺ T cells, NK cells, and neutrophils, respectively. Macrophages and monocytes were depleted using the Standard Macrophage Depletion Kit (Clodrosome® + Encapsome®), containing clodronate and control liposomes. Isotype control antibodies (rat IgG2a, mouse IgG2a) were obtained from BioLegend. All depleting antibodies were administered one day prior to both the first and last treatments. Tumor growth was monitored by visual inspection and palpation. Tumor size was measured with calipers, and tumor volume was calculated using the formula: V = width × length × (width + length)/2. Mice were sacrificed on day 14 after tumor inoculation.

### Flow cytometry

Tumors were collected from inoculated mice, and single-cell suspensions were prepared. Cells were washed with PBS and stained with Zombie Yellow™ Fixable Viability reagent (BioLegend) at 4 °C for 10 min to identify viable cells. To minimize nonspecific antibody binding, cells were incubated with anti-CD16/32 (S17011E, BioLegend) for 10 min. Cells were then washed with FACS buffer (PBS containing 0.5% FBS) and centrifuged at 300 × g for 5 min. After Fc receptor blocking, cells were resuspended in FACS buffer and stained with surface marker antibodies for 30 min at 4 °C. The following surface antibodies were used: BV510-CD45 (30-F11, BioLegend), PE-Cy7-CD45 (30-F11, BioLegend), BV480-CD8 (53-6.7, BD), BV570-CD4 (RM4-5, BioLegend), APC-Cy7-NK1.1 (PK136, BioLegend), BV421-CD11c (N418, BioLegend), BV711-CD11b (M1/70, BioLegend), BV785-Ly6C (HK1.4, BioLegend), Pacific Blue-Ly6G (1A8, BioLegend), PE-F4/80 (BM8, BioLegend), and PerCP-MHCII (M5/114.15.2, BioLegend). Following surface staining, cells were fixed using Fixation Buffer (BioLegend) and permeabilized with Permeabilization Buffer (eBioscience) for 10 min each. Intracellular staining was performed for 30 min at room temperature using the following antibodies: FITC-Granzyme B (GB11, BioLegend) and Alexa Fluor® 700-IFNγ (XMG1.2, BioLegend).

### *In vitro* transfection and analysis of protein expression

HEK293 or B16F10 cells were seeded in 24-well plates at a density of 1 × 10⁵ cells per well in DMEM supplemented with 10% fetal bovine serum, with a total volume of 1 mL per well. At 24 h post-seeding, cells were transfected in triplicate with 1 μg of LNP/pCBBGr99, LNP/pOX40L, or LNP/p4-1BBL, respectively. The cells were then incubated for 3 days at 37 °C in a humidified atmosphere with 5% CO₂. After incubation, transfected cells were harvested and stained with PE-conjugated anti-OX40L (RM134L, BioLegend) or PE-conjugated anti-4-1BBL (TKS-1, BioLegend), followed by analysis on a flow cytometer (Cytek Biosciences). For pCBBGr99-transfected cells, lysates were prepared using cell lysis buffer (Promega) for 10 min on ice. Following centrifugation at 600 × g for 5 min, 50 μL of supernatant was transferred to a 96-well white plate and mixed with 50 μL of luciferase substrate solution (Promega). Bioluminescence was recorded using an Orion L microplate luminometer (Berthold Detection System).

### Transcriptomic analysis

Anesthetized mice were subcutaneously inoculated in the left flank with 1 × 10⁵ B16F10 cells suspended in 0.1 mL serum-free DMEM. On day 7, anesthetized mice were administered 50 μL of a solution containing 1000 fmol LNP/pNC by intratumoral injection. The tumor was harvested the next day and immediately placed in RNAlater solution (Thermo Fisher Scientific). After RNA isolation, RNA sequencing analysis was performed by the Taiwan Genome Industry Alliance.

### *In vitro* DNA sensing inhibition

RAW264.7 cells (Bioresource Collection and Research Center, Taiwan; BCRC-60001) were cultured in DMEM supplemented with 10% (v/v) heat-inactivated fetal bovine serum at 37 °C under 5% CO₂. Cells were harvested and seeded in 24-well plates in quadruplicate at a density of 1 × 10⁶ cells/mL per well. After 2 h of culture, cells were treated with the following inhibitors or controls and incubated at 37 °C for 1 h: PBS, 10 μL DMSO (vehicle control), 100 μM Thiodigalactoside, 5 μM Andrographolide, 100 μM PF-06928215, 100 μM Hydroxychloroquine, 0.05 μM Quinacrine, 0.1 μM 9-amino-6-chloro-2-methoxyacridine, and 2 μM H-151. Subsequently, cells were stimulated with 1000 fmol LNP/pNC at 37 °C for 4 h. Cell culture supernatants were collected and analyzed for cytokine levels.

### Cytokine analysis

C57BL/6 mice (6-8 weeks old) were anesthetized and intramuscularly injected with 50 μL of 20 mM Tris-HCl (pH 7.0), empty LNP, pNC, or LNP/pNC. Plasma samples were collected at 4 and 24 h post-injection. Cytokine levels in the plasma were analyzed using uncoated ELISA kits for mouse TNFα, IL-6, and IFNγ (all from Invitrogen), according to the manufacturer's instructions. Briefly, 100 μL of capture antibody was added to each well and incubated overnight at 4 °C. After removing unbound antibody, wells were washed three times with PBS containing 0.05% Tween-20 and then blocked with 1× diluent (Invitrogen) for 1 h at room temperature. Subsequently, 100 μL of diluted plasma or supernatant was added to each well and incubated for 1 h at room temperature. After washing, 100 μL of detection antibody was added and incubated for 2 h at room temperature. Wells were then washed again, followed by the addition of streptavidin-HRP or avidin-HRP, which was incubated for 30 min at room temperature. After a final wash, TMB substrate (Clinical Science Product Inc.) was added and allowed to develop for 30 min. The reaction was stopped with sulfuric acid (H₂SO₄), and absorbance was measured at 450 nm using an ELISA reader.

### Statistical analysis

All data were analyzed using GraphPad Prism software (Version 10.3.1). Differences between the means of two experimental groups were assessed using a two-tailed unpaired t-test. For comparisons among multiple groups, one-way ANOVA followed by Tukey's multiple comparisons test was performed. A p-value of < 0.05 was considered statistically significant; 'ns' indicates no significance. Immune cell numbers in tumor tissues were log₁₀-transformed prior to correlation analysis.

## Results

### Plasmid DNA formulated in a lipid nanoparticle is stable at 4 ^°^C

**Figure 1:**
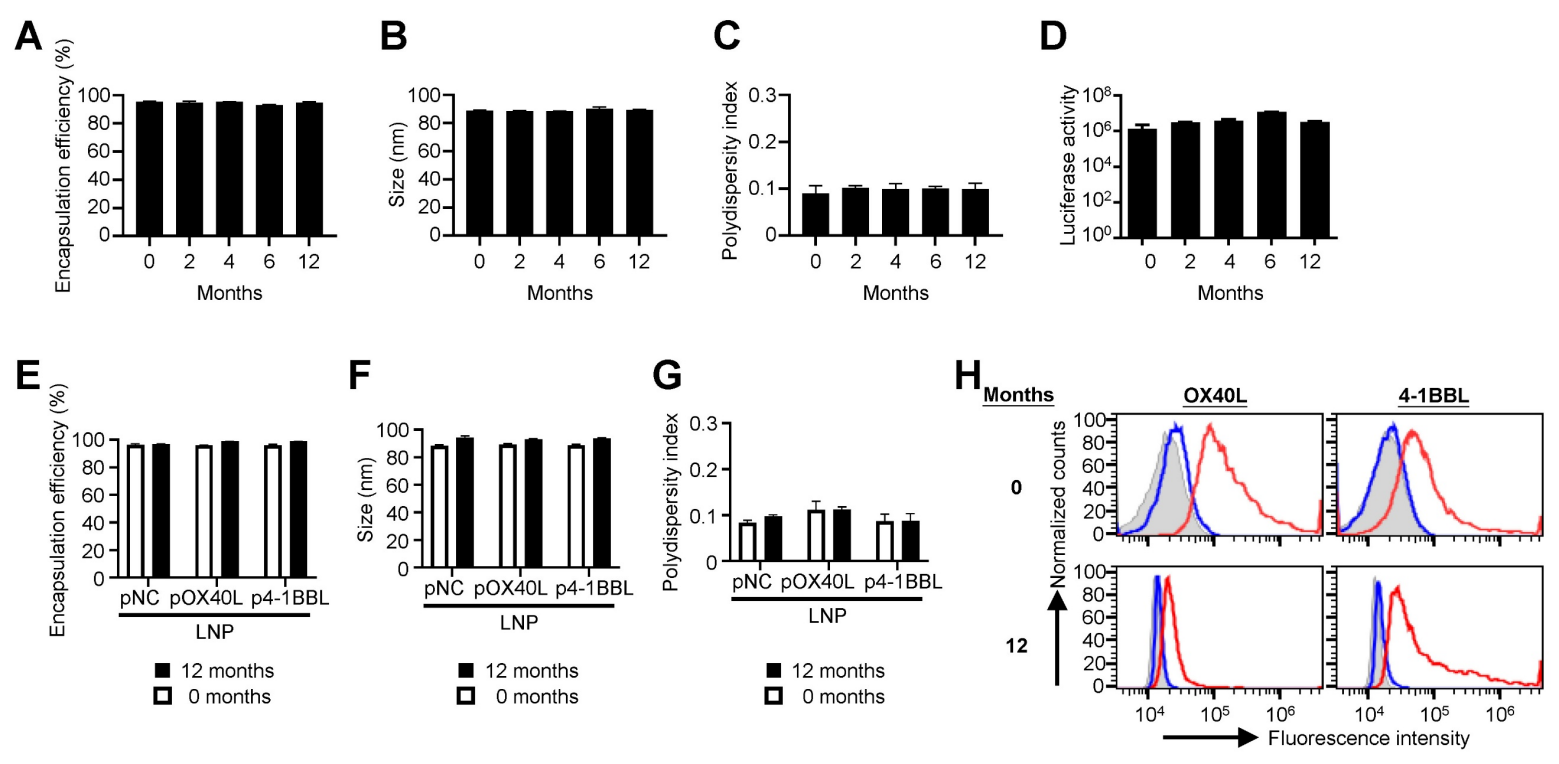
** Characterization of lipid nanoparticles (LNPs) encapsulating plasmids.** The pCBGr99, pOX40L, and p4-1BBL plasmids encode luciferase, OX40L, and 4-1BBL, respectively. Non-coding plasmid DNA (pNC) was used as a control. Plasmids were encapsulated in LNPs, and their encapsulation efficiency, particle size, and polydispersity index were analyzed for LNP/pCBGr99 (A-C), pNC, LNP/pOX40L, and LNP/p4-1BBL (E-G) at different time points as indicated. After transfection, luciferase activity in HEK293 cells (D) was assessed using a microplate luminometer, while the expression of encoded proteins in B16F10 cells (H) was analyzed by flow cytometry. The gray area represents pNC-transfected cells, the blue line denotes the isotype control, and the red line represents cells stained with anti-OX40L-PE or anti-4-1BBL-PE antibodies.

To evaluate the stability of pDNA encapsulated in LNPs, pCBGr99 plasmid encoding luciferase protein was formulated into LNPs. The LNPs were composed of cholesterol, 1,2-distearoyl-sn-glycero-3-phosphocholine (DSPC), 8-[(2-hydroxyethyl)[6-oxo-6-(undecyloxy)hexyl]amino]-octanoic acid, 1-octylnonyl ester (SM-102), and 1,2-dimyristoyl-rac-glycero-3-methoxy-PEG-2000 (DMG-PEG) at a molar ratio of 38.5:10:50:1.5. The LNPs encapsulating pCBGr99 were stored at 4 °C. Encapsulation efficiency (EE%) (Figure 1A), particle size (Figure 1B), and polydispersity index (PDI) (Figure 1C) were monitored at 2, 4, 6, and 12 months. Over the course of one year, EE%, particle size, and PDI remained stable at 93.1-95.4%, 88.3-90.1 nm, and ~0.1, respectively, when stored at 4 °C. Notably, LNP/pCBGr99 successfully transfected 293T cells, resulting in the expression of functional luciferase protein with comparable luciferase activity (Figure 1D).

To further examine the stability of other plasmids encapsulated in LNPs, plasmid DNA encoding OX40L (pOX40L), 4-1BBL (p4-1BBL), and control noncoding plasmid DNA (pNC) were formulated into LNPs. Consistent with LNP/ pCBGr99, the EE% (Figure 1E), particle size (Figure 1F), and PDI (Figure 1G) of LNP/pOX40L, LNP/p4-1BBL, and LNP/pNC remained stable when stored at 4 °C. Importantly, LNP/pOX40L and LNP/p4-1BBL successfully transfected B16F10 cancer cells, resulting in the expression of OX40L and 4-1BBL, respectively, even after 12 months of storage (Figure 1H). These findings suggest that pDNA formulated in LNPs is stable and retains functional transfection ability over extended storage periods.

### Treatment of LNP/pDNA induces antitumor effects in tumor-bearing mice

**Figure 2:**
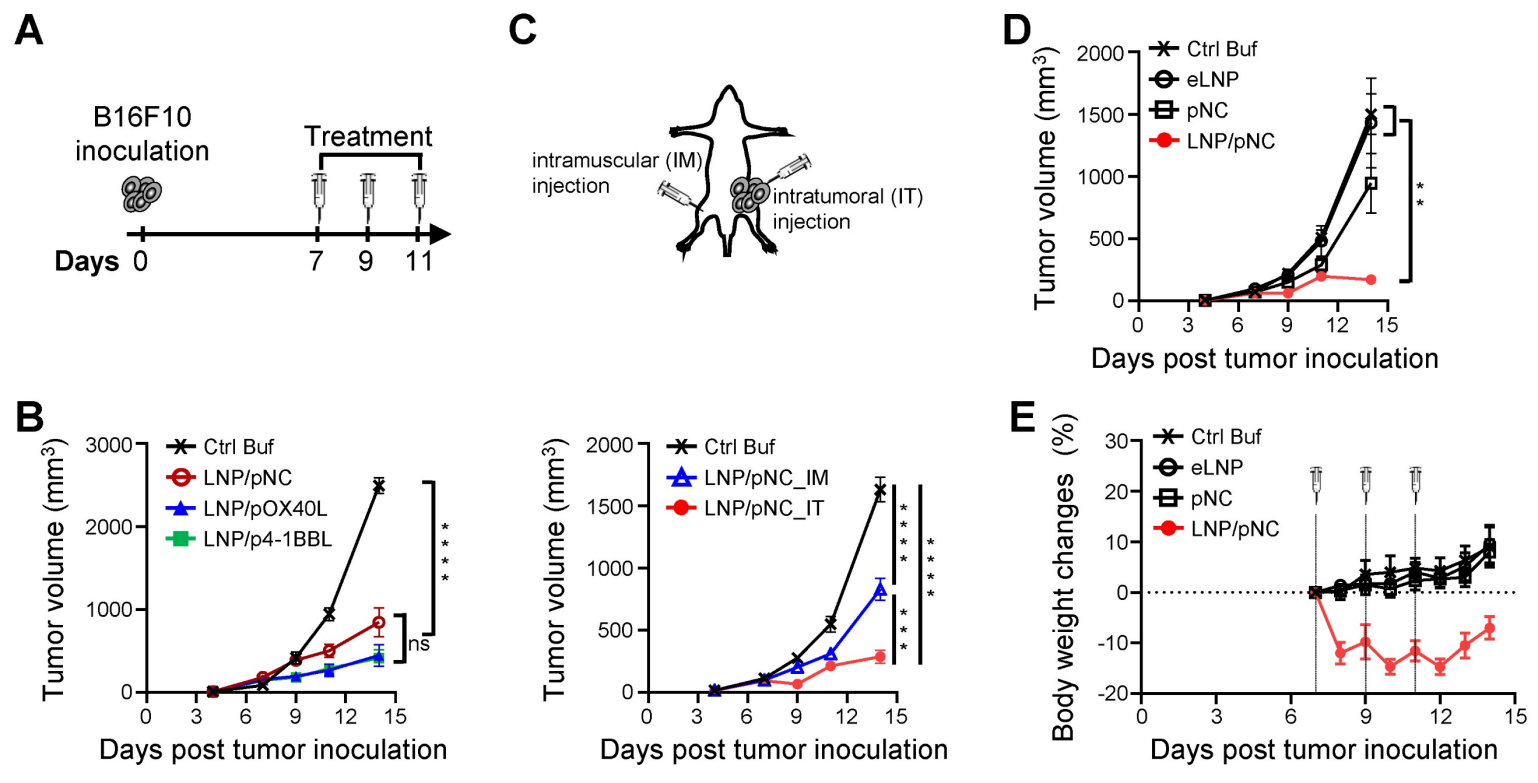
** Plasmid DNA-loaded LNPs effectively suppress tumor growth.** (A) C57BL/6 mice (6-8 weeks old) were inoculated with 1 × 10⁵ B16F10 cells in the left flank. Seven days later, tumor-bearing mice received treatment three times every two days. (B) Mice (n = 8 per group) were treated with control buffer (Ctrl Buf), LNP/pNC, LNP/pOX40L, or LNP/p4-1BBL, each equivalent to 100 femtomoles (fmole) of pDNA per dose. (C) Mice (n = 8 per group) received Ctrl Buf or LNP/pNC either locally via intratumoral (IT) injection or distally via intramuscular (IM) injection in the right hind leg, each equivalent to 1000 fmole pDNA per dose. (D) Mice (n = 7 per group) were treated with Ctrl Buf, empty LNP (eLNP), pNC, or LNP/pNC, each equivalent to 1000 fmole pDNA per dose. (E) Body weight changes (%) relative to the day of the first treatment are plotted. The tumor volume was calculated as: length × width × width/2 (mm^3^). Data are presented as means ± SEM. The statistical significance was determined using the one-way ANOVA with Tukey's multiple comparisons test. ****, P < 0.0001; ***, P < 0.001; **, P < 0.01.

Building on the successful transfection of B16F10 cancer cells with LNP/pOX40L and LNP/p4-1BBL *in vitro*, we further confirmed that OX40L and 4-1BBL were expressed in tumor cells following intratumoral injection of LNP/pOX40L and LNP/p4-1BBL, respectively, as determined by flow cytometry analysis (Figure S1). We next evaluated the therapeutic potential of LNPs carrying DNA encoding immunostimulatory proteins. Groups of C57BL/6 mice were inoculated subcutaneously with 1×10^5^ B16F10 cells. LNP/pOX40L or LNP/p4-1BBL was intratumorally injected into tumor-bearing mice on days 7, 9, and 11 post-inoculations (Figure 2A). Control buffer (Ctrl Buf) and LNP/pNC treatments were used as controls. Significant reductions in tumor growth were observed in all LNP-treated groups compared to the Ctrl Buf group (Figure 2B). The percentage of tumor growth inhibition ([1-tumor volume in the treatment group/tumor volume in the Ctrl Buf group] × 100%) on day 14 in LNP/pNC, LNP/pOX40L, and LNP/p4-1BBL were 66%, 82%, and 83%, respectively. Although LNP/pOX40L and LNP/p4-1BBL treatments induced greater tumor growth inhibition than LNP/pNC treatment, the differences were not statistically significant. These results suggest that treatment of tumor-bearing mice with LNP/pNC alone can inhibit tumor growth and play a crucial role. Therefore, we further focus on the antitumor effects induced by LNP/pNC.

Having demonstrated that intratumoral injection of LNP/pNC suppressed tumor growth, we next evaluated its systemic antitumor effects by examining the impact on distant, untreated tumors. Mice were inoculated with B16-F10 tumor cells on the left flank and treated with LNP/pNC either locally via intratumoral (IT) injection (LNP/pNC_IT) or distally via intramuscular (IM) injection in the right hind leg (LNP/pNC_IM). Both IT and IM administration of LNP/pDNA significantly inhibited tumor growth compared to the Ctrl Buf group, showing robust antitumor effects. Notably, IT administration consistently achieved greater tumor suppression than IM administration, with more pronounced tumor volume reductions observed in the IT LNP/pNC groups (Figure 2C). The percentage of tumor growth inhibition on day 14 in LNP/pNC_IM and LNP/pNC_IT were 49% and 82%, respectively. These findings underscore the efficacy of LNP/pNC in suppressing tumor progression and emphasize the superior potency of local (IT) delivery over systemic (IM) administration for antitumor therapy.

The antitumor activity of LNP/pNC can stem from the pNC, from the LNP, or from both components. To address this, we prepared LNPs containing pNC or empty LNP (eLNP). Tumor-bearing mice treated with LNP/pNC exhibited significantly reduced tumor growth compared to the Ctrl Buf group, eLNP group, and pNC group (plasmid DNA alone). Tumor volumes in the LNP/pNC-treated mice were consistently lower throughout the observation period, demonstrating a strong antitumor effect. In contrast, neither the eLNP nor the pNC group showed a significant impact on tumor growth compared to the Ctrl Buf group. These findings suggest that the antitumor activity is primarily due to the combined effects of pNC and the LNP formulation, rather than from either component alone (Figure 2D).

In addition, the body weights of mice in the Ctrl Buf group showed a steady increase. Similarly, the body weights of mice treated with eLNP and pNC followed a comparable upward trend. In contrast, mice that received LNP/pNC injections experienced a dramatic loss in body weight after each injection, followed by recovery over time (Figure 2E). To further assess safety, additional toxicological evaluations were performed. Mice were randomly assigned and intramuscularly injected with LNP/pNC in the left hind leg on days 0, 2, and 4. One week (LNP/pNC_1W) or thirteen weeks (LNP/pNC_13W) after the last injection, mice were sacrificed for toxicological evaluation. Mice treated with PBS (Control) served as references (Figure S2A). Except for alkaline phosphatase (ALP), which was transiently elevated at 1 week after LNP/pNC injection but returned to basal levels at 13 weeks, other serum biochemical indexes (aspartate aminotransferase, alanine aminotransferase, albumin, blood urea nitrogen, creatinine, and total bilirubin) showed no significant changes (Figure S2B). Importantly, hematological (Figure S2C) and histopathological (Figure S2D) analyses revealed no significant differences between LNP/pNC_1W or LNP/pNC_13W groups and the control. These preliminary safety assessments suggest that LNP/pNC injections may induce only transient and acute side effects.

### LNP/pNC induces transient inflammation

**Figure 3:**
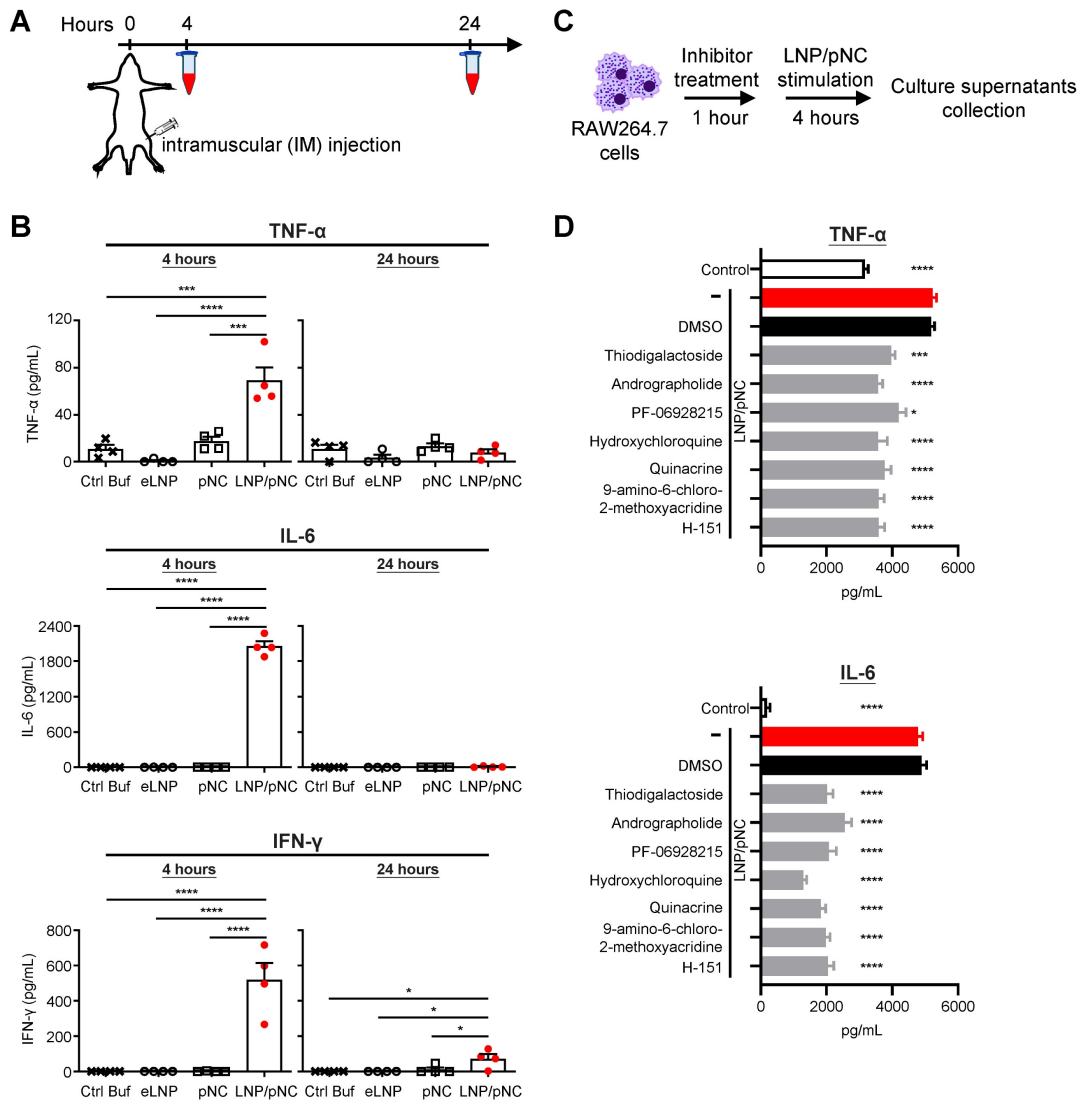
** Injection of LNP/pNC induces transient inflammatory responses.** (A) C57BL/6 mice (n = 4 per group) were treated with control buffer (Ctrl Buf), empty LNP (eLNP), pNC, or LNP/pNC, each equivalent to 1000 fmole pDNA. Plasma samples were collected at 4 and 24 h after injection. (B) Levels of TNF-α, IL-6 and IFN-γ in the plasma were determined by ELISA. (C) RAW264.7 cells were pretreated with specific inhibitors 1 h before exposure to LNP/pNC. Culture supernatants were collected 4 h after LNP/pNC treatment. (D) Levels of TNF-α and IL-6 were measured by ELISA. Data are presented as means ± SEM. The statistical significance was determined using the one-way ANOVA with Tukey's multiple comparisons test. ****, P < 0.0001; ***, P < 0.001; *, P < 0.05.

LNP-based mRNA delivery has been reported to induce severe inflammation 19, 34-36. Given that LNP/pNC injections caused body weight loss, we aimed to investigate whether LNP-formulated DNA also elicits inflammatory responses. Mice were intramuscularly injected with Ctrl Buf, eLNP, pNC, or LNP/pNC (Figure 3A). In mice treated with LNP/pNC, plasma levels of TNF-α, IL-6, and IFN-γ were significantly elevated at 4 h post-injection. By 24 h post-injection, cytokine levels had declined and returned to baseline, except for IFN-γ, which remained elevated (Figure 3B). These results are consistent with the findings that only mice in the LNP/pNC-treated group exhibited tumor growth inhibition (Figure 2D) and body weight loss (Figure 2E).

To elucidate the signaling pathways involved in LNP/pNC-induced cytokine production, RAW264.7 macrophages were pretreated with selective inhibitors prior to stimulation. Culture supernatants were collected 4 h after treatment, and TNF-α and IL-6 levels were quantified by ELISA (Figure 3C). As shown in Figure 3D, the untreated control group displayed baseline cytokine levels, whereas treatment with LNP/pNC alone markedly increased TNF-α and IL-6 production. DMSO-treated cells served as the vehicle control and exhibited cytokine levels comparable to the LNP/pNC-only group. All selective inhibitors tested significantly reduced TNF-α and IL-6 levels relative to the LNP/pNC-only group. Thiodigalactoside, an inhibitor of galectin-mediated interactions 37, significantly decreased cytokine production, suggesting the involvement of galectins. Andrographolide, a known inhibitor of absent in melanoma 2 (AIM2) inflammasome activation 38, also markedly reduced cytokine levels, highlighting a role for AIM2 signaling. PF-06928215, hydroxychloroquine, quinacrine, and 9-amino-6-chloro-2-methoxyacridine have been reported as cyclic GMP-AMP synthase (cGAS) inhibitors 39-42. Treatment with these compounds significantly decreased TNF-α and IL-6 production, implicating cGAS-associated mechanisms. H-151, a potent and selective small-molecule inhibitor of STING 43, also significantly suppressed cytokine secretion, indicating the involvement of STING.

To further validate these findings, we performed additional experiments using bone marrow-derived dendritic cells (BMDCs) and observed similar cytokine inhibition patterns when cells were pretreated with selective pathway inhibitors prior to stimulation, consistent with the results obtained in RAW264.7 cells (Figure S3). Furthermore, we found that cGAS, STING, and galectin-9 expression levels were increased in LNP/pNC-treated tumor tissues compared with controls. Although AIM2 expression was not detected in either control or LNP/pNC-treated tumor tissues, this may be attributable to the intrinsically low expression level of AIM2 in these samples (Figure S4). Collectively, these findings suggest that LNP/pNC-induced cytokine production is mediated by multiple signaling pathways, including those involving galectins, AIM2, cGAS, and STING.

### Intratumor injection of LNP/pDNA inhibits tumor growth via IFN signaling pathways

**Figure 4:**
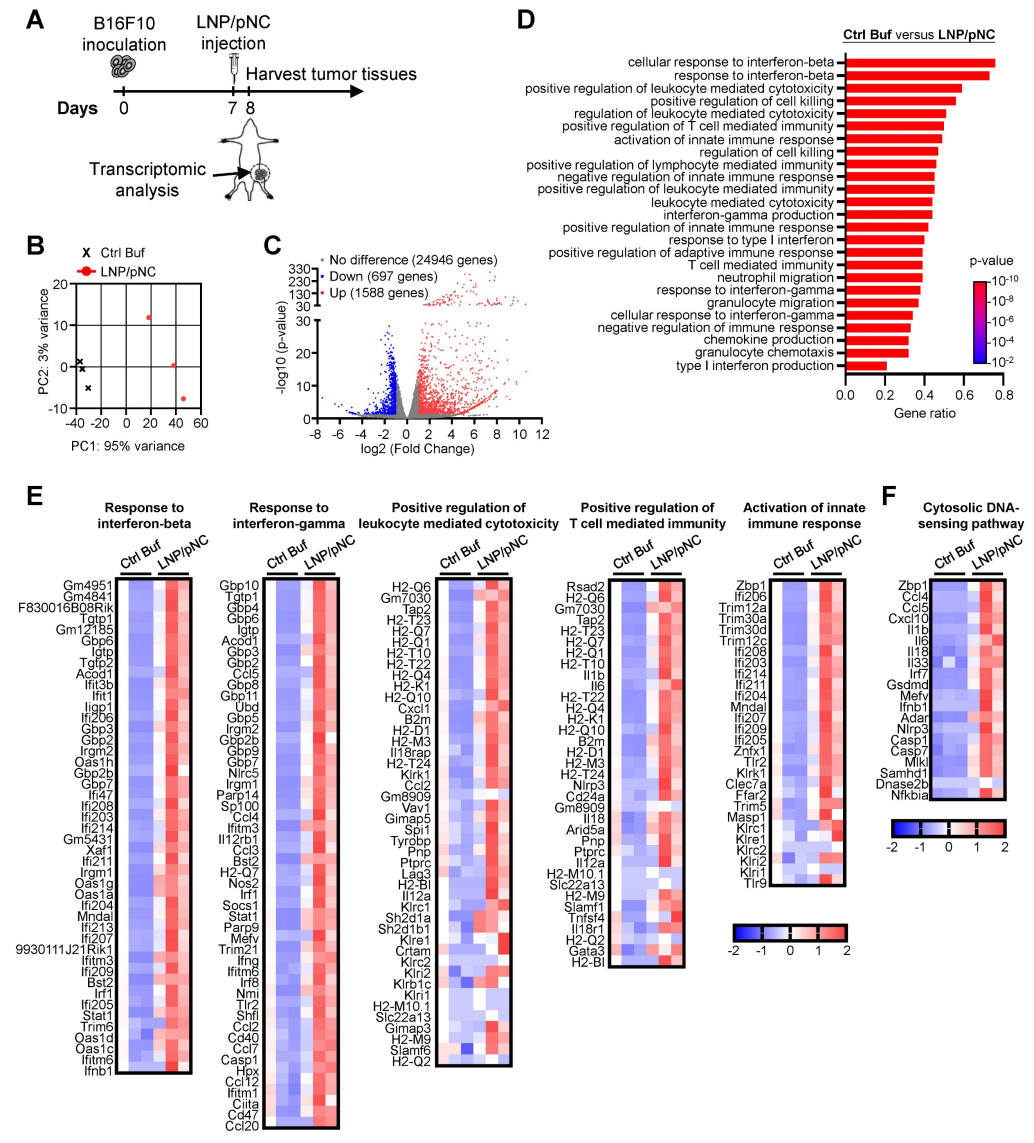
** Transcriptomic analysis of tumors following LNP/pNC treatment.** (A) C57BL/6 mice (6-8 weeks old) were inoculated with 1 × 10⁵ B16F10 cells in the left flank. Seven days later, tumor-bearing mice were treated with LNP/pNC (1000 femtomoles pDNA per dose). Mice were sacrificed on day 8, and tumors were excised for RNA sequencing analysis. Differential gene expression was assessed in tumors from LNP/pNC-treated mice (n = 3) compared with those from control buffer-treated mice (n = 3). (B) Principal component analysis (PCA) illustrating global differences in gene expression profiles. (C) Volcano plot showing -log₁₀(p-value) versus log₂(fold change) for all detected genes. (D) Top 25 pathways were identified by gene ontology enrichment analysis in tumors from LNP/pNC-treated mice compared with control buffer-treated mice. (E) Heatmaps showing genes involved in key immune-related pathways, including response to interferon-β, response to interferon-γ, positive regulation of leukocyte-mediated cytotoxicity, positive regulation of T cell-mediated immunity, and activation of innate immune response. (F) Heatmap of genes involved in cytosolic DNA-sensing pathways.

To decipher the impact of the LNP/pNC-induced inflammation on the tumor microenvironment, we performed transcriptomic analysis on the tumor tissues. Tumor-bearing mice were injected with LNP/pNC or Ctrl Buf seven days after tumor inoculation, and tumor tissues were collected for transcriptomic analysis one day following treatment (Figure 4A). A principal components analysis of tumor tissues showed LNP/pNC-treated mice and Ctrl Buf-treated mice formed separate clusters along principal components 1 and 2 (Figure 4B), providing evidence of their distinct transcriptome profile. There were 1588 upregulated genes and 697 downregulated genes (Figure 4C). Gene ontology enrichment analysis revealed that the top 25 biological process pathways were strongly associated with interferon responses, chemotaxis, immune regulation, inflammation, and cytotoxic activity. Among these, seven pathways were directly related to interferon responses, and five were associated with cytotoxic responses (Figure 4D). Importantly, we observed increased expression of several genes involved in these pathways. Heatmaps of representative pathways, including responses to interferon-β and interferon-γ, positive regulation of leukocyte-mediated cytotoxicity, positive regulation of T cell-mediated immunity, and activation of innate immune responses, are shown in Figure 4E. Additionally, we observed increased expression of genes involved in the cytosolic DNA-sensing pathway following LNP/pNC treatment (Figure 4F). These findings are consistent with the observation that specific inhibitors of the cytosolic DNA-sensing pathway reduce cytokine production (Figure 3D).

**Figure 5:**
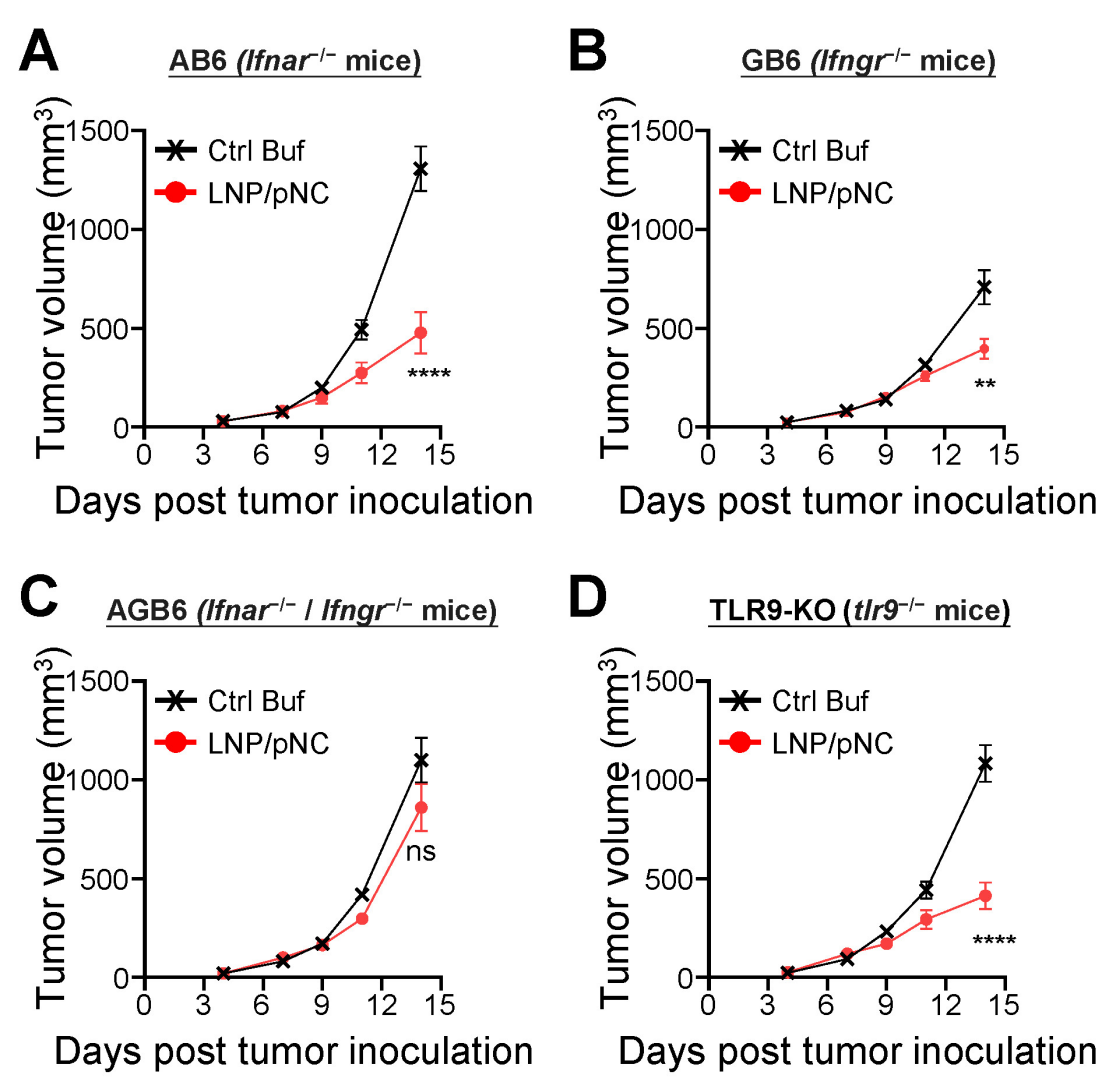
** Plasmid DNA-loaded LNPs suppress tumor growth via interferon-α/β and interferon-γ signaling pathways**. (A) AB6 (*Ifnar*^-/-^ mice), (B) GB6 (*Ifngr*^-/-^ mice), (C) AGB6 (*Ifnar*^-/-^ /* Ifngr*^-/-^ mice), and (D) TLR9-KO mice (*tlr9*^-/-^ mice) were inoculated with 1 × 10⁵ B16F10 cells in the left flank. Seven days later, tumor-bearing mice were treated with LNP/pNC (1000 femtomoles pDNA per dose) three times every two days. Mice treated with control buffer were included as reference controls. The tumor volume was calculated as: length × width × width/2 (mm^3^). Data are presented as means ± SEM. The statistical significance was determined using the unpaired t test. ****, P < 0.0001; **, P < 0.01.

Results obtained from gene ontology enrichment analysis (Figure 4D) suggest that interferon signaling pathways are involved in mediating tumor growth inhibition. To evaluate their contribution to the therapeutic efficacy of LNP/pNC, we utilized AB6 (*Ifnar*^-/-^ mice), GB6 (*Ifngr*^-/-^ mice), and AGB6 (*Ifnar*^-/-^ /* Ifngr*^-/-^ mice) mice. These mice were subcutaneously inoculated with 1 × 10⁵ B16-F10 cells and treated with LNP/pNC or control buffer on days 7, 9, and 11 post-inoculation, as outlined in Figure 2A. Treatment with LNP/pNC in AB6 (Figure 5A) or GB6 (Figure 5B) mice continued to inhibit tumor growth. However, this effect was lost in AGB6 mice (Figure 5C). These findings indicate that both interferon-α/β and interferon-γ signaling pathways are critical for the therapeutic efficacy of LNP/pNC.

Plasmids containing unmethylated cytosine-phosphate-guanine (CpG) motifs can bind to TLR9, leading to the activation of immune responses 44. To determine whether the TLR9 signaling pathway mediates the inhibition of tumor growth in LNP/pNC treated mice, TLR9-KO mice (*tlr9*^-/-^ mice) were utilized. As shown in Figure 5D, treatment with LNP/pNC in TLR9-KO mice were still to inhibit tumor growth. These results indicate that the therapeutic efficacy of LNP/pNC can occur independently of the TLR9 signaling pathway.

### Intratumor injection of LNP/pNC inhibits tumor growth by modulating tumor-infiltrating lymphocytes

**Figure 6:**
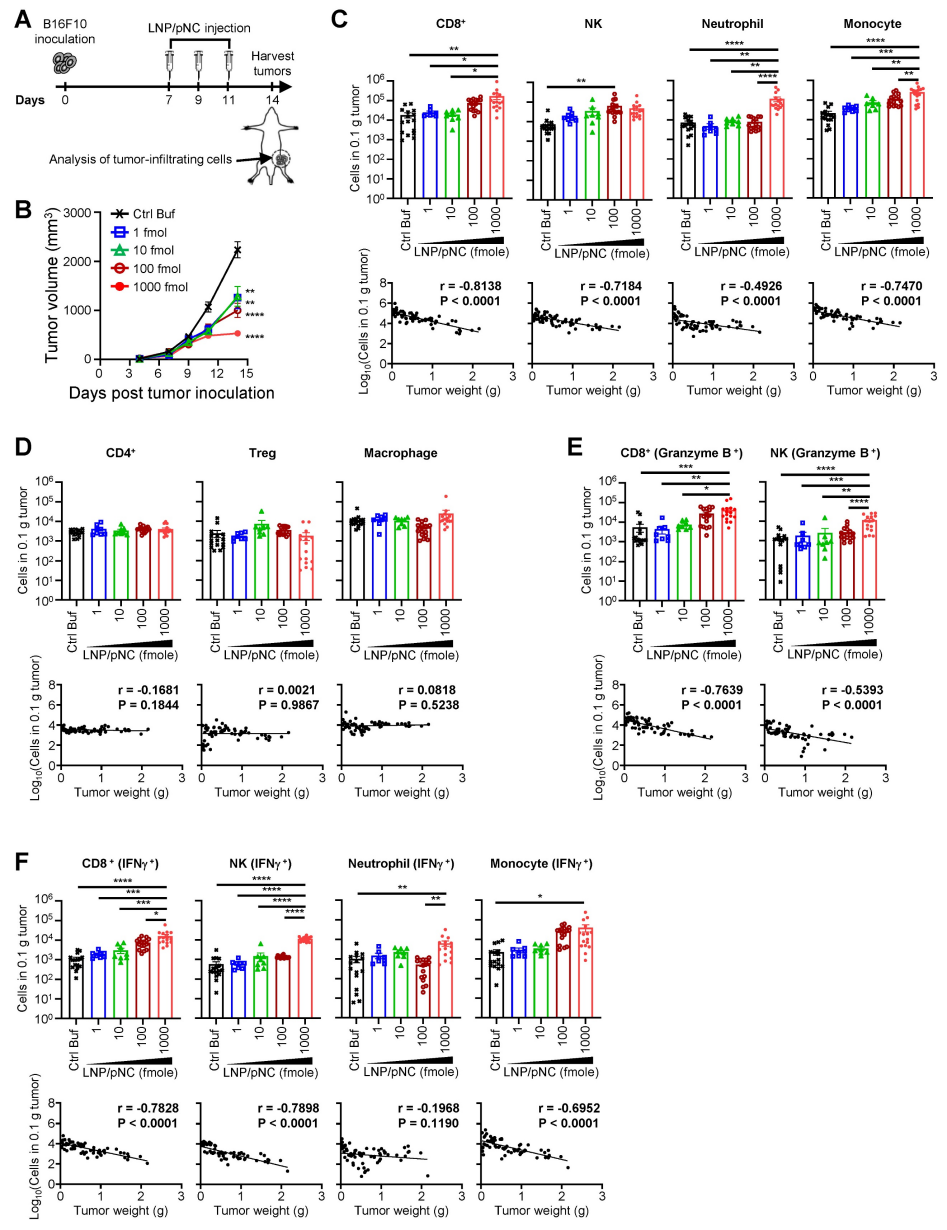
** Plasmid DNA-loaded LNPs modulate immune cell infiltration in tumors.** (A) C57BL/6 mice (6-8 weeks old) were inoculated with 1 × 10⁵ B16F10 cells in the left flank. Seven days later, tumor-bearing mice received control buffer or various LNP/pNC dosages treatment three times every two days. (B) The tumor volume was calculated as: length × width × width/2 (mm^3^). Data are presented as means ± SEM. (C-F) Mice were sacrificed on day 14, and tumors were excised and weighed. Single-cell suspensions were prepared, stained and analyzed by flow cytometry (upper panels). The cell numbers were logarithmically transformed before performing Pearson correlation analyses. Each Pearson correlation coefficient (r) and P value is shown in the upper right corner (lower panels). The statistical significance was determined using the one-way ANOVA with Tukey's multiple comparisons test. ****, P < 0.0001; ***, P < 0.001; **, P < 0.01; *, P < 0.05. The results of immune cell infiltration are obtained from two experiments.

We examined the effects of LNP/pNC at different dosages on tumor growth and immune effector cells within the tumor microenvironment 3 days after the last intratumoral injection (Figure 6A). Tumor growth inhibition correlated with the dosage of LNP/pNC, with higher dosages resulting in more pronounced antitumor effects (Figure 6B). Intratumoral injection of LNP/pNC increased the frequency of CD8^+^ T cells, NK cells, neutrophils, and monocytes (Figure 6C, the upper panel) as increased the dosage of LNP/pNC. The increase in these immune cells was associated with tumor growth inhibition (Figure 6C, lower panel). In contrast, the levels of CD4^+^ T cells, regulatory T cells, and macrophages remained unchanged across different dosages of LNP/pNC treatment (Figure 6D, upper panel) and showed no correlation with tumor growth inhibition (Figure 6D, lower panel). Moreover, the LNP/pNC induced cytotoxic granzyme B^+^ expression in CD8^+^ T cells and NK cells (Figure 6E, upper panel). The capacity of CD8^+^ T cells, NK cells, neutrophils, and monocytes to produce IFN-γ (Figure 6F, upper panel) were also enhanced by LNP/pNC. These results suggest that CD8^+^ T cells, NK cells, neutrophils, and monocytes may contribute induced tumor growth inhibition by LNP/pNC. These granzyme B- or IFN-γ-expressing cells were associated with tumor growth inhibition, except for IFN-γ-expressing neutrophils (Figure 6E and 6F, lower panel).

**Figure 7:**
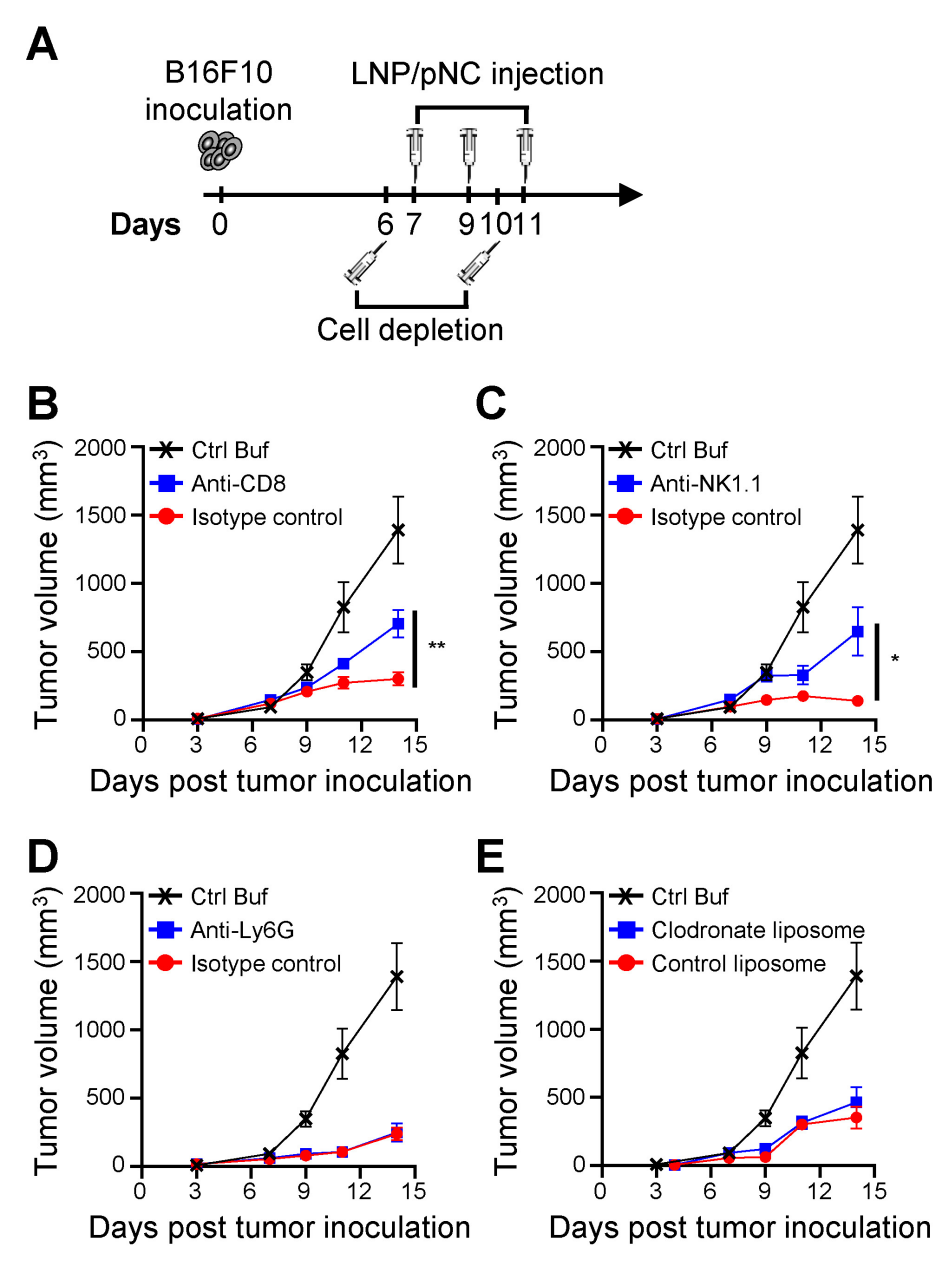
** Plasmid DNA-loaded LNPs inhibit tumor growth through CD8^+^ and NK cell-mediated immune responses.** (A) C57BL/6 mice (6-8 weeks old) were inoculated with 1 × 10⁵ B16F10 cells in the left flank. Seven days later, tumor-bearing mice were treated with LNP/pNC (1000 femtomoles pDNA per dose) three times every two days. Mice (n = 8 per group) were depleted of specific immune cell populations using (B) anti-CD8 antibodies for CD8⁺ T cells, (C) anti-NK1.1 antibodies for NK cells, (D) anti-Ly6G antibodies for neutrophils, and (E) clodronate liposomes for monocytes/macrophages. Corresponding isotype control antibodies or control liposomes were administered as appropriate. Mice treated with control buffer alone and without depletion served as reference controls. The tumor volume was calculated as: length × width × width/2 (mm^3^). Data are presented as means ± SEM. The statistical significance was determined using the unpaired t test. **, P < 0.01; *, P < 0.05.

Next, we investigated whether CD8^+^ T cells, NK cells, neutrophils, and monocytes are directly required for the therapeutic efficacy of LNP/pNC. To achieve this, anti-CD8, anti-NK1.1, and anti-Ly6G-depleting antibodies, as well as clodronate-encapsulated liposomes, were administered one day before both the first and last LNP/pNC treatments to deplete CD8^+^ T cells, NK cells, neutrophils, and monocytes, respectively (Figure 7A). Depletion of CD8^+^ T cells (Figure 7B) or NK cells (Figure 7C) partially abolished the therapeutic efficacy of LNP/pNC. In contrast, depletion of neutrophils (Figure 7D) or monocytes (Figure 7E) had no significant effect on LNP/pNC's therapeutic efficacy. These results indicate that the therapeutic efficacy of LNP/pNC is dependent on CD8^+^ T cells and NK cells.

## Discussion

In this study, we demonstrate that LNPs encapsulating various plasmid constructs—including those not encoding immunostimulatory proteins—effectively inhibit tumor growth (Figure 2B). Notably, all tested LNP/pDNAs maintained physicochemical stability (size, polydispersity, and encapsulation efficiency) and functional transfection capacity following long-term storage at 4 °C (Figure 1). These results provide a robust foundation for the development of LNP/pDNA-based immunotherapies with extended shelf life and sustained bioactivity. Compared to LNP-formulated mRNA, which is highly labile and often requires ultra-cold storage conditions 16, 25, LNP/pDNA formulations offer superior stability and scalability for clinical translation. This enhanced storage stability of LNP/pDNA not only improves logistical feasibility but also broadens potential applications in global health settings where cold-chain maintenance is limited.

The lack of antitumor activity observed with either naked pDNA or eLNPs highlights the necessity of both components for effective therapeutic response, underscoring a combination mechanism between the DNA cargo and the LNP delivery vehicle (Figure 2D). Notably, only LNP/pNC treatment elicited robust systemic cytokine responses—including TNF-α, IL-6, and IFN-γ—accompanied by transient body weight loss, indicative of acute innate immune activation driven by the LNP-mediated delivery of pDNA (Figure 3B and 2E). There is a consensus that LNP-formulated nucleic acids can facilitate cellular uptake 16. Effective nucleic acid delivery by LNPs depends on the successful release of the cargo into the cytosol—a process known as endosomal escape 45-48. Cytosolic plasmid DNA (double-stranded DNA) acts as a danger signal and is recognized by AIM2 and cGAS, triggering inflammatory responses 49-52. This notion is further supported by inhibitor studies showing reduced cytokine production (Figure 3D) and increased expression of genes involved in cytosolic DNA-sensing pathways (Figure 4F). These results are also consistent with recent studies using different LNP formulations 53, 54.

Critically, IT and IM administration of LNP/pNC in tumor-bearing mice elicited significant tumor growth inhibition, with IT delivery producing a more pronounced effect (Figure 2C). These results suggest that LNP/pNC treatment can induce systemic antitumor effects. Despite its potency, intratumoral injection requires direct access to tumor lesions. This limitation can be addressed using image-guided techniques, such as ultrasound or computed tomography, to accurately target tumor sites 26. More importantly, local administration of LNP/pNC also conferred systemic protection and induced tumor regression at distal sites.

Transcriptomic analysis of tumor tissues reveals that IT administration of LNP/pNC effectively reprograms the tumor microenvironment from 'cold' to 'hot.' Genes associated with interferon signaling, leukocyte-mediated cytotoxicity, and the activation of both adaptive and innate immune responses were upregulated following LNP/pNC treatment (Figure 4). The functional relevance of these pathways was confirmed *in vivo*. Tumor suppression by LNP/pNC was abrogated in *Ifnar*^-/-^ / *Ifngr*^-/-^ double knockout mice (AGB6), but not in single knockout mice. In these experiments, tumor cells retained interferon responsiveness, whereas non-tumor (host) cells lacked interferon signaling capacity. These findings suggest that both type I and type II interferon signaling in host cells are independently required for the therapeutic efficacy of LNP/pNC (Figure 5). Furthermore, immunophenotyping of tumor-infiltrating lymphocytes following LNP/pNC treatment revealed increased infiltration of CD8⁺ T cells, NK cells, neutrophils, and monocytes in a dose-dependent manner. Notably, granzyme B and IFN-γ expression in CD8⁺ and NK cells were strongly associated with tumor growth inhibition (Figure 6). Depletion studies functionally validated the requirement of CD8⁺ T cells and NK cells—but not neutrophils or monocytes—for LNP/pNC-mediated tumor suppression, underscoring the importance of cytotoxic lymphocytes in driving therapeutic responses (Figure 7).

While our findings highlight the potent immunostimulatory and antitumor effects of LNP/pNC, this study has some limitations. First, only one formulation was used in the current study, which cannot be considered representative of all LNPs. Some critical factors such as the structural characteristics of ionizable lipids and other formulation parameters may significantly influence the delivery performance of LNPs. In addition, certain ionizable lipids exhibit intrinsic adjuvant activity 15, 55, 56, the precise relationship between LNP composition, biodistribution, and immunogenicity warrants further investigation. Future studies incorporating lipid structure-function analyses and *in vivo* tracking will help clarify how LNP formulations modulate immune activation and therapeutic efficacy. Second, since the RNA-seq data were derived from bulk tumor tissues, they do not resolve which specific cell populations are the primary responders to LNP/pNC stimulation. Future studies employing single-cell RNA sequencing, in situ hybridization, or cell-type-specific knockout models will be necessary to identify the key responder populations.

Together, these findings suggest that the immune activation induced by LNP/pNC is multifaceted, relying on both innate and adaptive immune mechanisms. The observed activation of DNA-sensing pathways, coupled with the induction of cytotoxic lymphocyte responses, presents a compelling rationale for further investigation of LNP/pDNA platforms in cancer immunotherapy. How to mitigate the transient side effects while preserving antitumor activity remains an important question for future research. Given the translational advantages of pDNA over mRNA, including cost-effective production and enhanced stability, LNP/pDNA holds significant promise as a next-generation nucleic acid therapeutic.

## Conclusion

Our results highlight the potential of lipid nanoparticle-formulated plasmid DNA (LNP/pDNA) as a stable, effective, and scalable immunotherapy platform. LNP/pDNA induces potent antitumor effects through the activation of DNA-sensing pathways and the recruitment of cytotoxic immune effectors, independent of encoded immunostimulatory proteins. These findings underscore the importance of the combined use of plasmid DNA and LNP carriers for therapeutic efficacy, supporting the continued development of LNP/pDNA systems for cancer immunotherapy and beyond.

## Supplementary Material

Supplementary figures.

## Abbreviations

AB6: *Ifnar*^-/-^ mice
AGB6: *Ifnar*^-/-^ / *Ifngr*^-/-^ mice
AIM2: absent in melanoma 2
cGAS: cyclic GMP-AMP synthase
CpG: cytosine-phosphate-guanine
Ctrl Buf: control buffer
DLS: dynamic light scattering
DMG-PEG: 1,2-dimyristoyl-rac-glycero-3-methoxy-PEG-2000
DMSO: dimethyl sulfoxide
DSPC: 1,2-distearoyl-sn-glycero-3-phosphocholine
EE: Encapsulation efficiency
ELISA: enzyme-linked immunosorbent assay
eLNP: empty LNP
GB6: *Ifngr*^-/-^ mice
IFN-γ: interferon-γ
IL-6: Interleukin 6
IM: intramuscular injection
IT: intratumoral injection
LNPs: lipid nanoparticles
mRNAs: messenger RNAs
p4-1BBL: plasmid DNA encoding 4-1BBL
pCBGr99: pVAX1-CBGr99 encodes luciferase
pDNAs: plasmid DNAs
PDI: polydispersity index
PEG: PolyethyleneGlycol
pNC: noncoding plasmid DNA
pOX40L: plasmid DNA encoding OX40L
SM-102: 8-[(2-hydroxyethyl)[6-oxo-6-(undecyloxy)hexyl]amino]-octanoic acid, 1-octylnonyl ester
STING: stimulator of interferon genes
TLR9: Toll-like receptor 9
KO: knockout
TNF-α: tumor necrosis factor

## References

[1] Chen DS, Mellman I (2017). Elements of cancer immunity and the cancer-immune set point. Nature.

[2] Melero I, Berman DM, Aznar MA, Korman AJ, Perez Gracia JL, Haanen J (2015). Evolving synergistic combinations of targeted immunotherapies to combat cancer. Nat Rev Cancer.

[3] Sharma P, Hu-Lieskovan S, Wargo JA, Ribas A (2017). Primary, Adaptive, and Acquired Resistance to Cancer Immunotherapy. Cell.

[4] Whiteside TL, Demaria S, Rodriguez-Ruiz ME, Zarour HM, Melero I (2016). Emerging Opportunities and Challenges in Cancer Immunotherapy. Clin Cancer Res.

[5] Belgrad J, Fakih HH, Khvorova A (2024). Nucleic Acid Therapeutics: Successes, Milestones, and Upcoming Innovation. Nucleic Acid Ther.

[6] Kulkarni JA, Witzigmann D, Thomson SB, Chen S, Leavitt BR, Cullis PR (2021). The current landscape of nucleic acid therapeutics. Nat Nanotechnol.

[7] Lou W, Zhang L, Wang J (2024). Current status of nucleic acid therapy and its new progress in cancer treatment. Int Immunopharmacol.

[8] Singh AK, Goel K, Dhanawat M (2025). Plasmid DNA and mRNA delivery: Approaches and challenges. Adv Immunol.

[9] Sun X, Setrerrahmane S, Li C, Hu J, Xu H (2024). Nucleic acid drugs: recent progress and future perspectives. Signal Transduct Target Ther.

[10] Anderson EJ, Rouphael NG, Widge AT, Jackson LA, Roberts PC, Makhene M (2020). Safety and Immunogenicity of SARS-CoV-2 mRNA-1273 Vaccine in Older Adults. N Engl J Med.

[11] Azeem M, Cancemi P, Mukhtar F, Marino S, Peri E, Di Prima G (2025). Efficacy and limitations of SARS-CoV-2 vaccines - A systematic review. Life Sci.

[12] Baden LR, El Sahly HM, Essink B, Kotloff K, Frey S, Novak R (2021). Efficacy and Safety of the mRNA-1273 SARS-CoV-2 Vaccine. N Engl J Med.

[13] Polack FP, Thomas SJ, Kitchin N, Absalon J, Gurtman A, Lockhart S (2020). Safety and Efficacy of the BNT162b2 mRNA Covid-19 Vaccine. N Engl J Med.

[14] Sahin U, Muik A, Derhovanessian E, Vogler I, Kranz LM, Vormehr M (2020). COVID-19 vaccine BNT162b1 elicits human antibody and T(H)1 T cell responses. Nature.

[15] Hassett KJ, Benenato KE, Jacquinet E, Lee A, Woods A, Yuzhakov O (2019). Optimization of Lipid Nanoparticles for Intramuscular Administration of mRNA Vaccines. Mol Ther Nucleic Acids.

[16] Hou X, Zaks T, Langer R, Dong Y (2021). Lipid nanoparticles for mRNA delivery. Nat Rev Mater.

[17] Liao HC, Shen KY, Yang CH, Chiu FF, Chiang CY, Chai KM (2024). Lipid nanoparticle-encapsulated DNA vaccine robustly induce superior immune responses to the mRNA vaccine in Syrian hamsters. Mol Ther Methods Clin Dev.

[18] Yang CH, Shen KY, Ho HM, Huang CY, Cheng YJ, Pu CC (2024). Boosting DNA vaccine power by lipid nanoparticles surface engineered with amphiphilic bioresorbable copolymer. Mol Ther Nucleic Acids.

[19] Ndeupen S, Qin Z, Jacobsen S, Bouteau A, Estanbouli H, Igyarto BZ (2021). The mRNA-LNP platform's lipid nanoparticle component used in preclinical vaccine studies is highly inflammatory. iScience.

[20] Alameh MG, Tombacz I, Bettini E, Lederer K, Sittplangkoon C, Wilmore JR (2021). Lipid nanoparticles enhance the efficacy of mRNA and protein subunit vaccines by inducing robust T follicular helper cell and humoral responses. Immunity.

[21] Amor NP, Guo K, Zhang S, Xia J, Yang Y, Lin A (2025). Lipid Nanoparticle: Beyond Delivery Vehicle-Unveiling Its Immunological Adjuvant Potential. FASEB J.

[22] Dai W, Xing M, Sun L, Lv L, Wang X, Wang Y (2024). Lipid nanoparticles as adjuvant of norovirus VLP vaccine augment cellular and humoral immune responses in a TLR9- and type I IFN-dependent pathway. J Virol.

[23] Zhou Y, Gao Z, He Z, Wen Z, Zhang Z, Jin X (2025). Nanoparticulate lipid adjuvants induce robust immunity against RSV infection. Science China Materials.

[24] Kawai A, Noda M, Hirata H, Munakata L, Matsuda T, Omata D (2024). Lipid Nanoparticle with 1,2-Di-O-octadecenyl-3-trimethylammonium-propane as a Component Lipid Confers Potent Responses of Th1 Cells and Antibody against Vaccine Antigen. ACS Nano.

[25] Pardi N, Hogan MJ, Porter FW, Weissman D (2018). mRNA vaccines - a new era in vaccinology. Nat Rev Drug Discov.

[26] Hong WX, Haebe S, Lee AS, Westphalen CB, Norton JA, Jiang W (2020). Intratumoral Immunotherapy for Early-stage Solid Tumors. Clin Cancer Res.

[27] Hutmacher C, Neri D (2019). Antibody-cytokine fusion proteins: Biopharmaceuticals with immunomodulatory properties for cancer therapy. Adv Drug Deliv Rev.

[28] Nestle FO, Farkas A, Conrad C (2005). Dendritic-cell-based therapeutic vaccination against cancer. Curr Opin Immunol.

[29] Yan Z, Zhang Z, Chen Y, Xu J, Wang J, Wang Z (2024). Enhancing cancer therapy: the integration of oncolytic virus therapy with diverse treatments. Cancer Cell Int.

[30] Hamouda AEI, Filtjens J, Brabants E, Kancheva D, Debraekeleer A, Brughmans J (2024). Intratumoral delivery of lipid nanoparticle-formulated mRNA encoding IL-21, IL-7, and 4-1BBL induces systemic anti-tumor immunity. Nat Commun.

[31] Liyanage W, Kannan G, Kannan S, Kannan RM (2025). Efficient Intracellular Delivery of CRISPR-Cas9 Ribonucleoproteins Using Dendrimer Nanoparticles for Robust Genomic Editing. Nano Today.

[32] Qin Y, Rouatbi N, Wang JT, Baker R, Spicer J, Walters AA (2024). Plasmid DNA ionisable lipid nanoparticles as non-inert carriers and potent immune activators for cancer immunotherapy. J Control Release.

[33] Samaridou E, Heyes J, Lutwyche P (2020). Lipid nanoparticles for nucleic acid delivery: Current perspectives. Adv Drug Deliv Rev.

[34] Parhiz H, Brenner JS, Patel PN, Papp TE, Shahnawaz H, Li Q (2022). Added to pre-existing inflammation, mRNA-lipid nanoparticles induce inflammation exacerbation (IE). J Control Release.

[35] Sharma P, Hoorn D, Aitha A, Breier D, Peer D (2024). The immunostimulatory nature of mRNA lipid nanoparticles. Adv Drug Deliv Rev.

[36] Tahtinen S, Tong AJ, Himmels P, Oh J, Paler-Martinez A, Kim L (2022). IL-1 and IL-1ra are key regulators of the inflammatory response to RNA vaccines. Nat Immunol.

[37] van Hattum H, Branderhorst HM, Moret EE, Nilsson UJ, Leffler H, Pieters RJ (2013). Tuning the preference of thiodigalactoside- and lactosamine-based ligands to galectin-3 over galectin-1. J Med Chem.

[38] Gao J, Peng S, Shan X, Deng G, Shen L, Sun J (2019). Inhibition of AIM2 inflammasome-mediated pyroptosis by Andrographolide contributes to amelioration of radiation-induced lung inflammation and fibrosis. Cell Death Dis.

[39] Alarcon-Esposito J, Nagiri RK, Gan L, Sinha SC (2025). Identification and development of cGAS inhibitors and their uses to treat Alzheimer's disease. Neurotherapeutics.

[40] An J, Minie M, Sasaki T, Woodward JJ, Elkon KB (2017). Antimalarial Drugs as Immune Modulators: New Mechanisms for Old Drugs. Annu Rev Med.

[41] Ding C, Song Z, Shen A, Chen T, Zhang A (2020). Small molecules targeting the innate immune cGAS-STING-TBK1 signaling pathway. Acta Pharm Sin B.

[42] Hall J, Brault A, Vincent F, Weng S, Wang H, Dumlao D (2017). Discovery of PF-06928215 as a high affinity inhibitor of cGAS enabled by a novel fluorescence polarization assay. PLoS One.

[43] Haag SM, Gulen MF, Reymond L, Gibelin A, Abrami L, Decout A (2018). Targeting STING with covalent small-molecule inhibitors. Nature.

[44] Cornelie S, Hoebeke J, Schacht AM, Bertin B, Vicogne J, Capron M (2004). Direct evidence that toll-like receptor 9 (TLR9) functionally binds plasmid DNA by specific cytosine-phosphate-guanine motif recognition. J Biol Chem.

[45] Cavegn A, Waldner S, Wang D, Sedzicki J, Kuzucu EU, Zogg M (2025). Intracellular processing of DNA-lipid nanoparticles: A quantitative assessment by image segmentation. J Control Release.

[46] Chatterjee S, Kon E, Sharma P, Peer D (2024). Endosomal escape: A bottleneck for LNP-mediated therapeutics. Proc Natl Acad Sci U S A.

[47] Hoekstra D, Rejman J, Wasungu L, Shi F, Zuhorn I (2007). Gene delivery by cationic lipids: in and out of an endosome. Biochem Soc Trans.

[48] Schlich M, Palomba R, Costabile G, Mizrahy S, Pannuzzo M, Peer D (2021). Cytosolic delivery of nucleic acids: The case of ionizable lipid nanoparticles. Bioeng Transl Med.

[49] Dong M, Fitzgerald KA (2024). DNA-sensing pathways in health, autoinflammatory and autoimmune diseases. Nat Immunol.

[50] Hornung V, Ablasser A, Charrel-Dennis M, Bauernfeind F, Horvath G, Caffrey DR (2009). AIM2 recognizes cytosolic dsDNA and forms a caspase-1-activating inflammasome with ASC. Nature.

[51] Motwani M, Pesiridis S, Fitzgerald KA (2019). DNA sensing by the cGAS-STING pathway in health and disease. Nat Rev Genet.

[52] Sun L, Wu J, Du F, Chen X, Chen ZJ (2013). Cyclic GMP-AMP synthase is a cytosolic DNA sensor that activates the type I interferon pathway. Science.

[53] Patel MN, Tiwari S, Wang Y, O'Neill S, Wu J, Omo-Lamai S (2025). Safer non-viral DNA delivery using lipid nanoparticles loaded with endogenous anti-inflammatory lipids. Nat Biotechnol.

[54] Senapati S, Bertolini TB, Minnier MA, Yazicioglu MN, Markusic DM, Zhang R (2025). Inhibition of IFNAR-JAK signaling enhances tolerability and transgene expression of systemic non-viral DNA delivery. Mol Ther Nucleic Acids.

[55] Gao Z, Yang H, He Z, Zhou Y, Ge X, Liu H (2025). Cost-effective yet high-performance ionizable lipids for mRNA-lipid nanoparticle vaccines. Biomaterials.

[56] Yang H, Gao Z, Zhou Y, Ge X, Pan X, Li J (2025). Tertiary amine N-oxide zwitterionic lipids facilitate muscle-selective mRNA vaccine delivery for enhancing cDC1-mediated antitumor efficacy. J Control Release.

