# Supplementary material for: Lipid nanoparticle-formulated DNA acts as a potent immune modulator for cancer immunotherapy through interferon signaling pathways
Source: Theranostics. 2026 Jan 1;16(5):2342–56. doi: 10.7150/thno.121364 (PMC12712898; doi:10.7150/thno.121364)
Supplement: Supplementary file 1 — Supplementary figures. [file thnov16p2342s1.pdf]

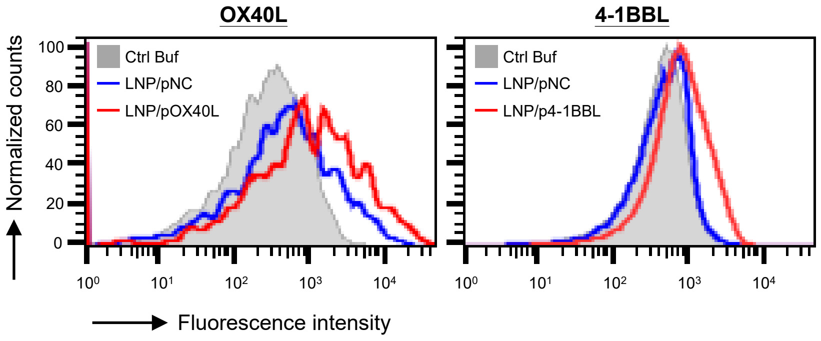

Figure S1. Expression of OX40L and 4-1BBL in tumors. C57BL/6 mice were inoculated with 1×10^5 B16F10 cells in the left flank. Seven days later, tumor-bearing mice received intratumoral injections of Ctrl Buf, LNP/pNC, LNP/pOX40L, or LNP/p4-1BBL, each equivalent to 1000 femtomoles (fmole) of pDNA per dose. Mice were sacrificed on day 10, and tumors were excised. Single-cell suspensions were prepared, stained with anti-CD45, anti-OX40L-PE, or anti-4-1BBL-PE antibodies, and analyzed by flow cytometry. $CD45^-$ cells were considered tumor cells and gated to assess OX40L or 4-1BBL expression. The gray area and blue line represent tumors injected with Ctrl Buf and LNP/pNC, respectively, while the red line represents tumors injected with LNP/pOX40L or LNP/p4-1BBL.

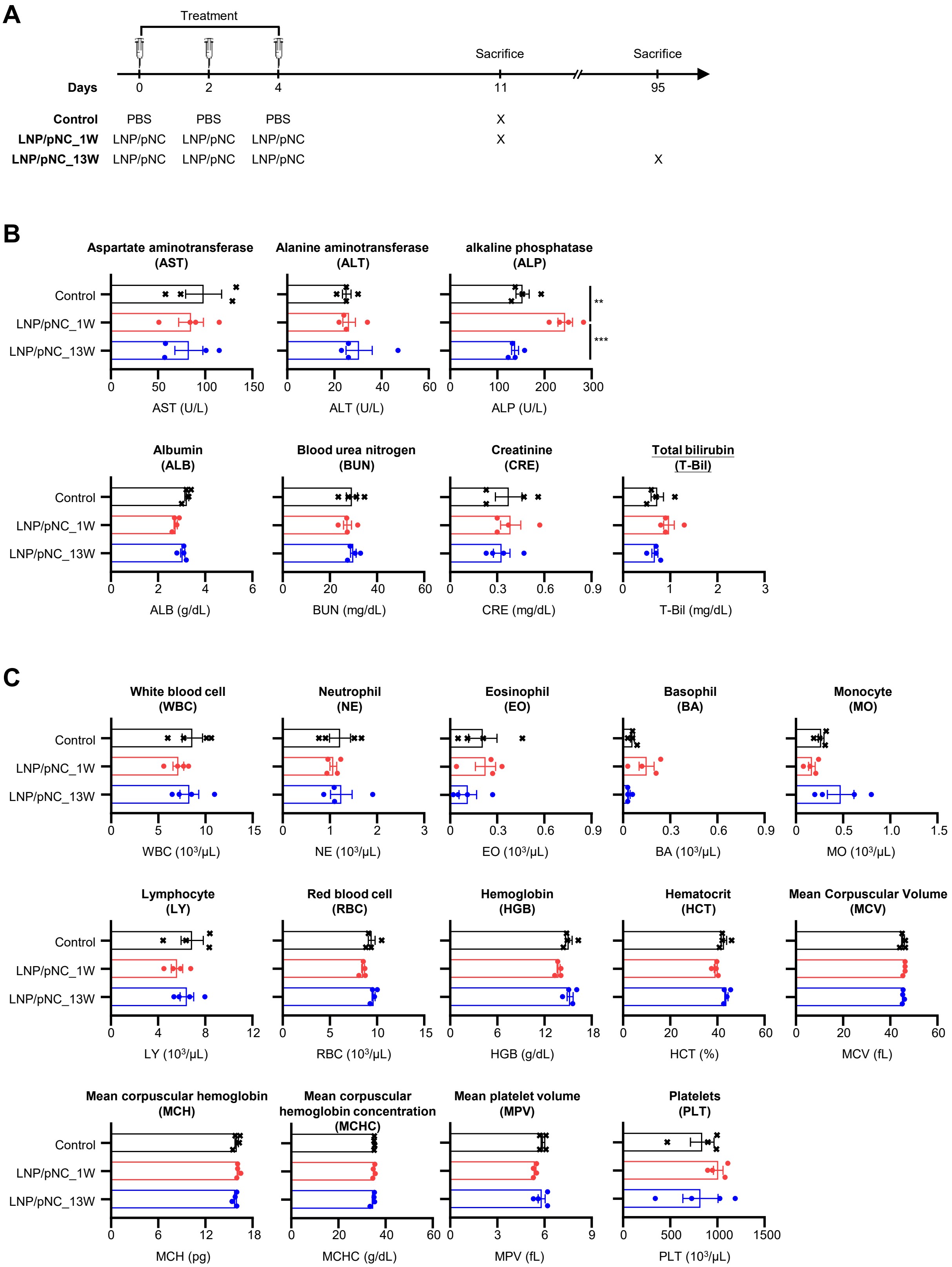
A
Treatment
Sacrifice
Sacrifice
Days
0
2
4
11
95
Control
PBS
PBS
PBS
X
LNP/pNC_1W
LNP/pNC
LNP/pNC
LNP/pNC
X
LNP/pNC_13W
LNP/pNC
LNP/pNC
LNP/pNC
X
B
Aspartate aminotransferase (AST)
Alanine aminotransferase (ALT)
alkaline phosphatase (ALP)
Control
LNP/pNC_1W
LNP/pNC_13W
**
***
AST (U/L)
ALT (U/L)
ALP (U/L)
Albumin (ALB)
Blood urea nitrogen (BUN)
Creatinine (CRE)
Total bilirubin (T-Bil)
ALB (g/dL)
BUN (mg/dL)
CRE (mg/dL)
T-Bil (mg/dL)
C
White blood cell (WBC)
Neutrophil (NE)
Eosinophil (EO)
Basophil (BA)
Monocyte (MO)
WBC (10³/μL)
NE (10³/μL)
EO (10³/μL)
BA (10³/μL)
MO (10³/μL)
Lymphocyte (LY)
Red blood cell (RBC)
Hemoglobin (HGB)
Hematocrit (HCT)
Mean Corpuscular Volume (MCV)
LY (10³/μL)
RBC (10³/μL)
HGB (g/dL)
HCT (%)
MCV (fL)
Mean corpuscular hemoglobin (MCH)
Mean corpuscular hemoglobin concentration (MCHC)
Mean platelet volume (MPV)
Platelets (PLT)
MCH (pg)
MCHC (g/dL)
MPV (fL)
PLT (10³/μL)

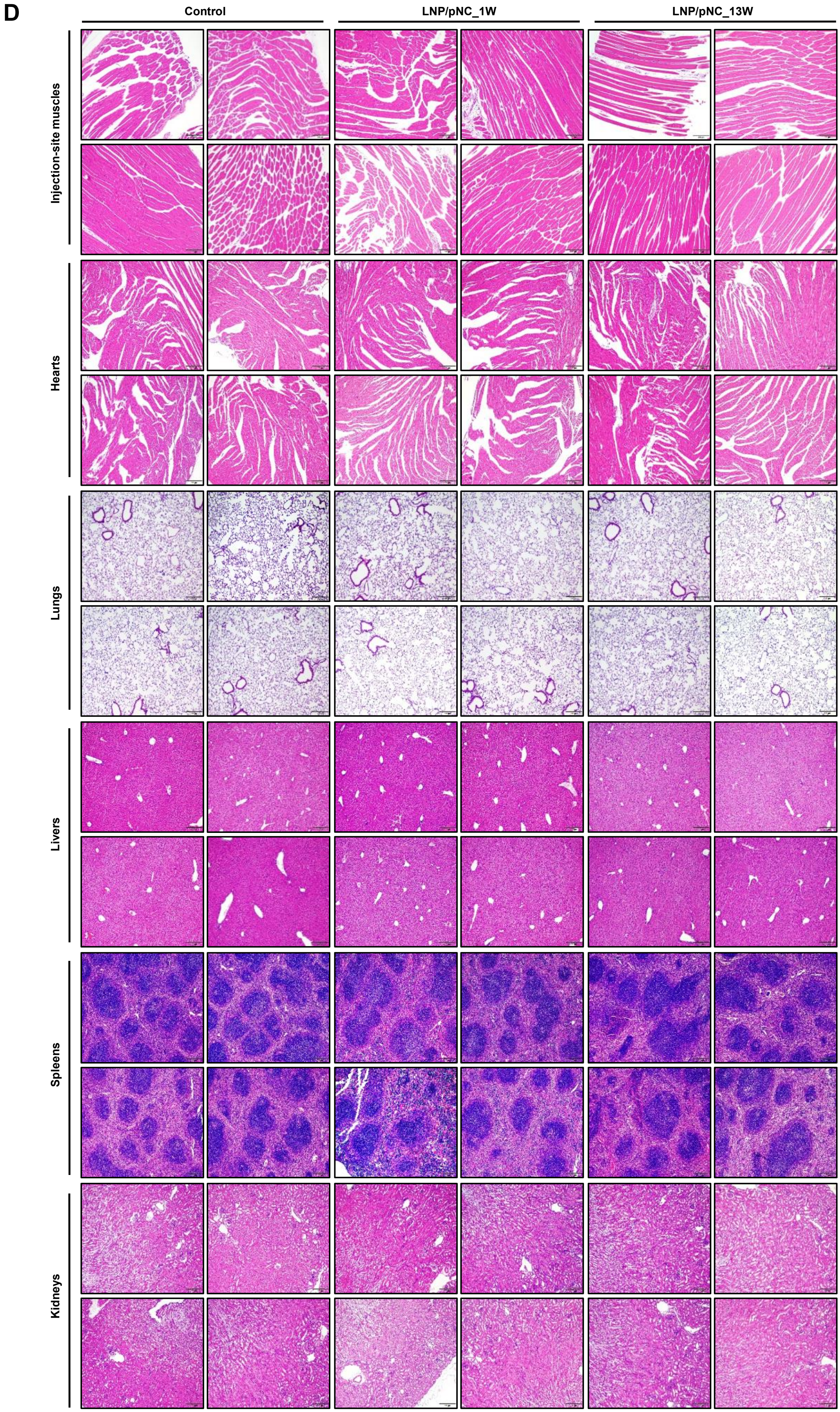
D
Control
LNP/pNC_1W
LNP/pNC_13W
Injection-site muscles
Hearts
Lungs
Livers
Spleens
Kidneys

Figure S2. Safety assessment of LNP/pNC. (A) C57BL/6 mice (n = 4 per group) were intramuscularly injected with LNP/pNC on days 0, 2, and 4, and sacrificed 1 week (LNP/pNC_1W) or 13 weeks (LNP/pNC_13W) after the last injection for toxicological evaluation. PBS-treated mice served as controls. Blood samples and organs from individual mice were collected at sacrifice. (B) Serum biochemical analytes were measured using FUJI DRI-CHEM slides and analyzed with the FUJI DRI-CHEM NX500i Chemistry System. The statistical significance was determined using the one-way ANOVA with Tukey's multiple comparisons test. **, $P < 0.01$; ***, $P < 0.001$. (C) Hematological assays were conducted using the Mindray BC-5000 Vet analyzer. (D) The injection site muscles, hearts, lungs, livers, spleens, and kidneys were subjected to histologic analysis by H & E staining. Scale bars represent 200 μm.

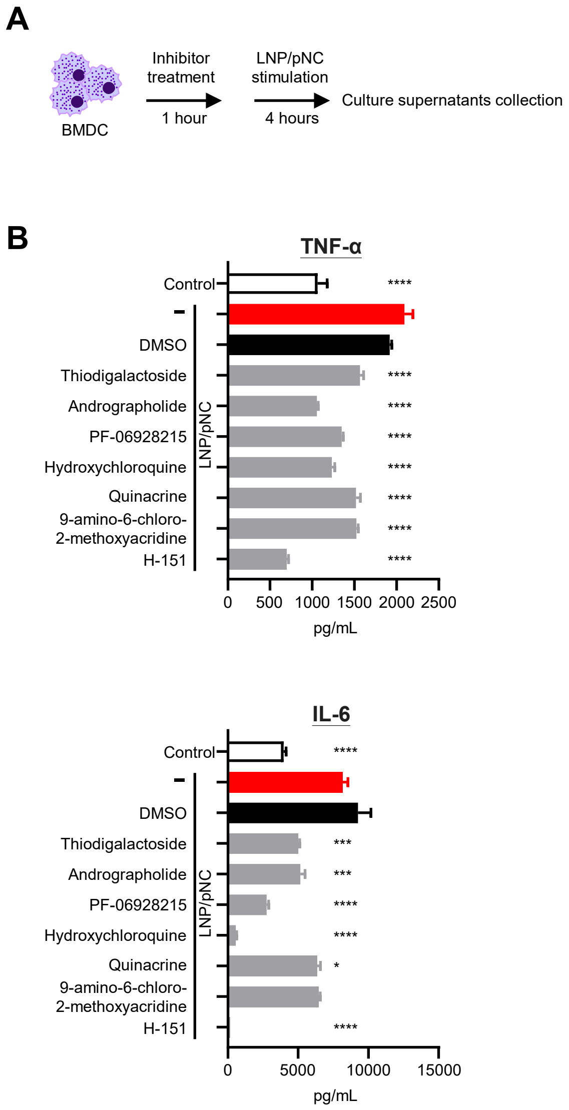

Figure S3. LNP/pNC-induced cytokine production in bone marrow–derived dendritic cells is mediated by galectin, AIM2, cGAS, and STING signaling pathways. (A) Bone marrow-derived dendritic cells (BMDCs) were pretreated with specific inhibitors 1 hour before exposure to LNP/pNC. Culture supernatants were collected 4 hours after LNP/pNC treatment. (B) Levels of TNF-α and IL-6 were measured by ELISA. Data are presented as means ± SEM. The statistical significance was determined using the one-way ANOVA with Tukey's multiple comparisons test. ****, $P < 0.0001$; ***, $P < 0.001$; *, $P < 0.05$.

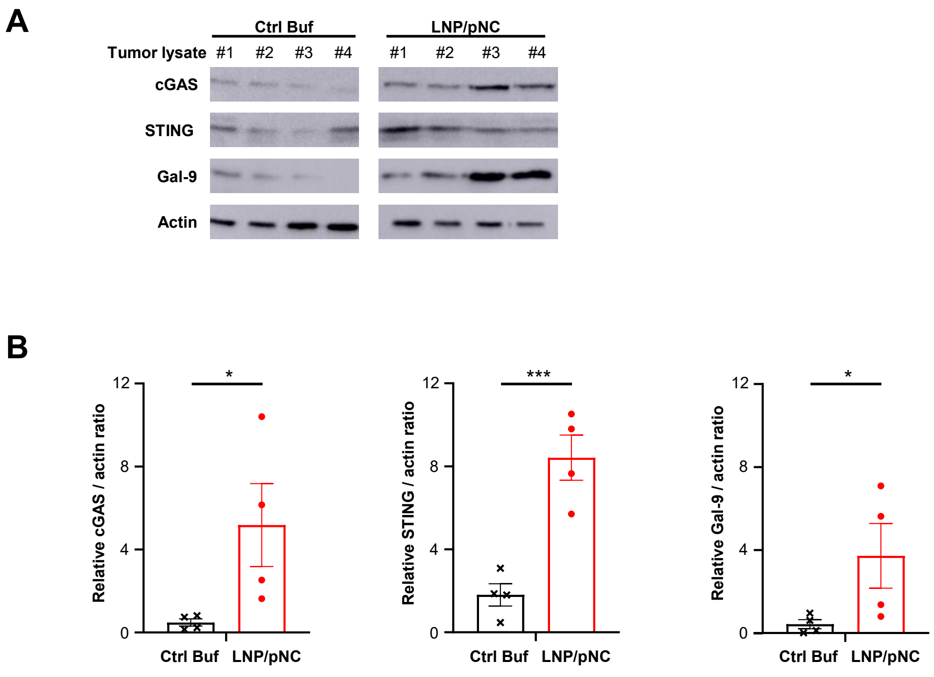

Figure S4. Expression of cGAS, STING, and Gal-9 in tumors. C57BL/6 mice (n = 4 per group) were inoculated with 1 × 10^5 B16F10 cells in the left flank. Seven days later, tumor-bearing mice received intratumoral injections of Ctrl Buf or LNP/pNC (equivalent to 1000 femtomoles of pDNA). Mice were sacrificed on day 10, and tumors were excised. Tumor lysates were prepared. (A) Expression of cGAS, STING, Gal-9, and actin in each tumor were examined by immunoblotting with specific antibodies. The actin expression level is served as internal control for each tumor lysate. (B) Relative expression of cGAS, STING, and Gal-9 to actin ratios were quantified by ImangeJ. Data are presented as means ± SEM. The statistical significance was determined using the unpaired t test. ***, $P < 0.001$; *, $P < 0.05$.
